# Environmental, Climatic, and Parasite Molecular Factors Impacting the Incidence of Cutaneous Leishmaniasis Due to *Leishmania tropica* in Three Moroccan Foci

**DOI:** 10.3390/microorganisms10091712

**Published:** 2022-08-25

**Authors:** Othmane Daoui, Hamza Bennaid, Mouad Ait Kbaich, Idris Mhaidi, Nacer Aderdour, Hassan Rhinane, Souad Bouhout, Khadija Akarid, Meryem Lemrani

**Affiliations:** 1 Laboratory of Parasitology and Vector-Borne-Diseases, Institut Pasteur du Maroc, Casablanca 20250, Morocco; 2 Molecular Genetics and Immunophysiopathology Research Team, Health and Environment Laboratory, Aïn Chock Faculty of Sciences, Hassan II University of Casablanca, Casablanca 20000, Morocco; 3 Information Retrieval and Data Analytics Laboratory, National School of Computer Science and System Analysis (Ensias), Rabat 10112, Morocco; 4 Geosciences Laboratory, Faculty of Sciences Ain Chock, Hassan II University of Casablanca, Casablanca 20000, Morocco; 5 Directorate of Epidemiology, Division of Infectious Diseases, Service of Parasitic Diseases, Ministry of Health of Morocco, Rabat 10020, Morocco

**Keywords:** cutaneous leishmaniasis, risk factors, incidence, *Leishmania tropica*, population structure

## Abstract

Cutaneous leishmaniasis (CL) occurring due to *Leishmania tropica* is a public health problem in Morocco. The distribution and incidence of this form of leishmaniasis have increased in an unusual way in the last decade, and the control measures put in place are struggling to slow down the epidemic. This study was designed to assess the impact of climatic and environmental factors on CL in *L. tropica* foci. The data collected included CL incidence and climatic and environmental factors across three Moroccan foci (Foum Jemaa, Imintanout, and Ouazzane) from 2000 to 2019. Statistical analyses were performed using the linear regression model. An association was found between the occurrence of CL in Imintanout and temperature and humidity (*r*^2^ = 0.6076, df = (1.18), *p*-value = 3.09 × 10^−5^; *r*^2^ = 0.6306, df = (1.18), *p*-value = 1.77 × 10^−5^). As a second objective of our study, we investigated the population structure of *L.* *tropica* in these three foci, using the nuclear marker internal transcribed spacer 1 (ITS1). Our results showed a low-to-medium level of geographic differentiation among the *L.* *tropica* populations using pairwise differentiation. Molecular diversity indices showed a high genetic diversity in Foum Jemaa and Imintanout; indeed, 29 polymorphic sites were identified, leading to the definition of 13 haplotypes. Tajima’s D and Fu’s F test statistics in all populations were not statistically significant, and consistent with a population at drift–mutation equilibrium. Further analysis, including additional DNA markers and a larger sample size, could provide a more complete perspective of *L. tropica*’s population structure in these three regions. In addition, further research is needed to better understand the impact of climatic conditions on the transmission cycle of *Leishmania*, allowing both for the development of effective control measures, and for the development of a predictive model for this parasitosis.

## 1. Introduction

Leishmaniasis is a vector-borne disease caused by an intracellular parasite of the genus *Leishmania*, which is transmitted by bloodsucking dipterans. The disease is among the top three vector-borne diseases, along with malaria and filariasis [1].

Cutaneous leishmaniasis (CL) is the most widespread and common form of the disease and causes three forms of skin lesions (ulcers, ulcero-crusted lesions, and nodular lesions) on exposed parts of the body, leaving lifelong scars and serious disability or stigma, especially among young women. The number of CL cases in the world is as high as one million, according to the World Health Organization [2].

In Morocco, three species of *Leishmania* are known to be causative agents of CL: the two most dominant species are *L*. *major* and *L*. *tropica*, the third species is *L*. *infantum*, which is less common than the other two *Leishmania* species. The distribution of CL due to *L*. *infantum* in Morocco is not well defined; it is frequently found in *L*. *tropica* foci and even in *L*. *major* foci in pre-Saharan areas [3].

Amongst the three clinically important *Leishmania* species, *L. tropica* has the largest geographic distribution and is considered to be a public health threat by the Moroccan Ministry of Health [4].

*L. tropica* was reported for the first time in the rural locality of Tanant (Azilal province, High Atlas) in 1987 [5]. Subsequently, *L. tropica* cases have increasingly been reported from different regions of the country; in 1991, Pratlong et al. [6] unveiled a vast *L. tropica* focus in central and southern areas such as Guelmim, Agadir, and Essaouira. Since that time and until today, the *L. tropica*-induced CL has continued to spread throughout the country, affecting Taza, Zouagha Moulay Yaacoub, Chichaoua, Settat, and Lbrouj [7,8,9,10]. There has also been an overlap and an integration of *L. tropica* in certain *L. infantum* foci, such as Ouazzane, and *L. major* foci, such as Toundout, Agdz, and Ouarzazate [11,12]. In Morocco, anthroponotic transmission is, so far, the only recognized mode, in spite of recordings of *L. tropica* infection in animal hosts [13]; therefore, the transmission cycle of *L. tropica* involves a vector *Phlebotomus sergenti*, which is essential for the emergence of the disease, and a vertebrate host, which is the human. *P. sergenti* is characterized by a wide geographical distribution in the Mediterranean basin; its density is significant in arid and Saharan zones, and it can be found in mid-to-high altitudes [13].

The pathogens, hosts, and vectors involved in the transmission cycle of vector-borne diseases are environmentally sensitive [14]. Leishmaniasis has been reported to be impacted by climate change and human modification of ecosystems [3].

Valero and Uriarte [15] divided risk factors into three categories: socioeconomic, environmental, and climatic variables; the latter showed significant associations with the incidence of both visceral leishmaniasis (VL) and CL. Socioeconomic factors were also associated with disease incidence in the vulnerable human populations of arid and tropical developing regions [15]. The conversion of natural forest to other land uses in the last few decades has led to habitat fragmentation and altered landscape composition [16]. The spread of the vector and disease at macroscales is associated with migration and the expansion of human populations into natural areas, the creation of roadways, energy networks, new farmlands, and poorly planned urban development [17,18,19,20]; climatic conditions are generally important risk factors for vector-borne diseases [21]. However, overall, it is not well understood how the interactions among all of the climatic, environmental, and socioeconomic factors influence the risk of leishmaniasis.

In Morocco, a few studies have been carried out to elucidate the impact of biotic and abiotic factors on the occurrence and repartition of CL [22,23]. However, it is difficult to make a definitive statement about the link between these risk factors and the epidemiology of leishmaniasis, especially CL, due to *L. tropica*, which continues to evolve in terms of both the number of human cases and the repartition area.

To contribute to a better understanding of the epidemiology of CL induced by *L. tropica* and the impact of climatic and environment factors on this form of leishmaniasis, we carried out a survey in three CL foci: Foum Jemaa, Imintanout, and Ouazzane. In addition to genotyping, we studied the population structure of *L. tropica* collected in the three regions, using the evolutionary marker internal transcribed spacer 1 (ITS1). We also investigated whether the mean temperature, annual rainfall, relative humidity, average wind speed, and vegetation index influenced the increase in leishmaniasis incidence in the three CL foci.

## 2. Materials and Methods

### 2.1. Study Area

**Figure 1:**
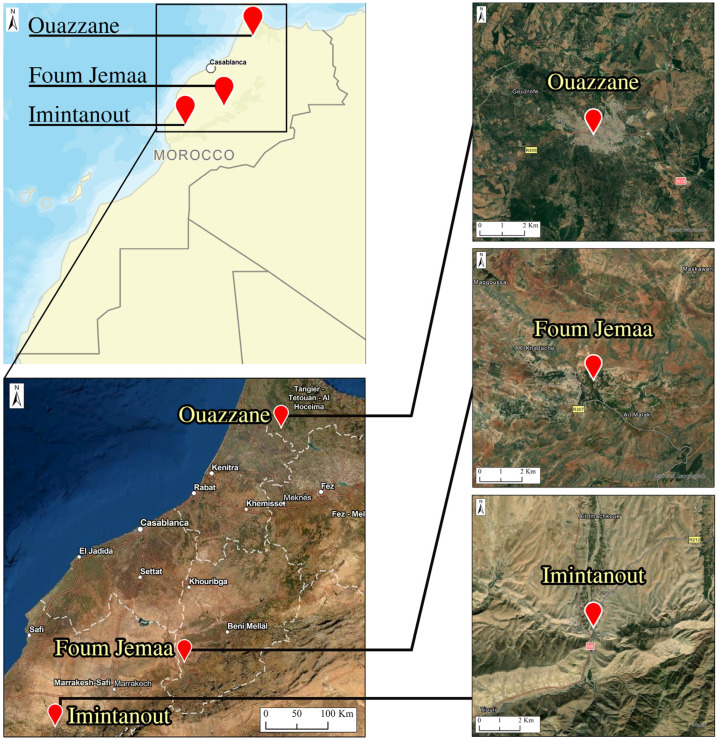
Geographical locations of the study areas.

This study enrolled patients originating from three regions known as CL endemic foci: the Foum Jemaa locality in the center of Morocco (31°57′52.03″ N, 6°59′26.22″ W) (744 m asl), High Atlas in Azilal Province; Imintanout village (31°10′13.90″ N, 8°50′45.86″ W) (912 m asl), located in the southwest of the country, in the province of Chichaoua; and the periphery of the city of Ouazzane (34°47′40.89″ N, 5°34′4.49″ W) (614 m asl), in northern Morocco (Figure 1).

### 2.2. Ethical Considerations

Informed consent was obtained from all adults who participated in the study. Consent for the inclusion of young children was obtained from parents or guardians. The study was reviewed and approved by the Ethical Review Committee for Biomedical Research (CERB) of the Faculty of Medicine and Pharmacy, Rabat, Morocco (IORG 0006594 FWA 00024287).

### 2.3. Patients and Sampling

During an active screening campaign for CL cases performed for 1 week each year between 2018 and 2020 in the three CL foci, samples were collected from 80 patients. All recruited patients presented skin lesions clinically suggestive of CL, and were never treated by Glucantime injection. Pregnant women and patients presenting chronic illness (e.g., blood pressure issues, diabetes, etc.) were not skin sampled.

For each consenting patient, before sampling, we acquired a completed structured questionnaire that included the relevant personal, epidemiological, and clinical data. 

After cleaning the lesions, a gentle scraping at the edge of the lesion was performed, and the collected skin scraping was spread on slide smears for amastigote detection. A cotton swab was also used for molecular studies, as described by Daoui, O et al. [11]. 

We cultured *Leishmania* on an RPMI 1640 medium (Biowest, Nuaille, France) supplemented with 2 mM l-glutamine (Eurobio, Les Ulis, France), 10% fetal bovine serum (Biowest, Nuaille, France), and 1% penicillin/streptomycin (100 U/mL penicillin and 100 μg/mL streptomycin; Biowest, Nuaille, France), followed by incubation at 25 °C.

### 2.4. Population at Risk

The population census estimation in each province was obtained from the Epidemiology and Disease Control Department, Moroccan Ministry of Health, for the period 2000–2019, except for Ouazzane, where the period was shorter (2009 to 2019) due to a lack of information, justified by the fact that CL cases in Ouazzane were very low and were, therefore, added to the closest province to Ouazzane. The annual incidence rate of CL in each province was defined as follows: incidence rate = (total number of CL cases per year/total population at risk) × 100,000 (Table S1).

### 2.5. Meteorological Parameters

The meteorological data were obtained from the National Meteorology Department of Morocco and from TuTiempo.net (powered by Tutiempo Network, S.L., Madrid, Spain), which is a low-cost, citizen-based PM sensor network system that has been deployed globally. TuTiempo.net offers measurements of climate data and meteorological parameters (temperature, rainfall, humidity, and wind speed) using satellite images.

### 2.6. Normalized Difference Vegetation Index

**Figure 2:**
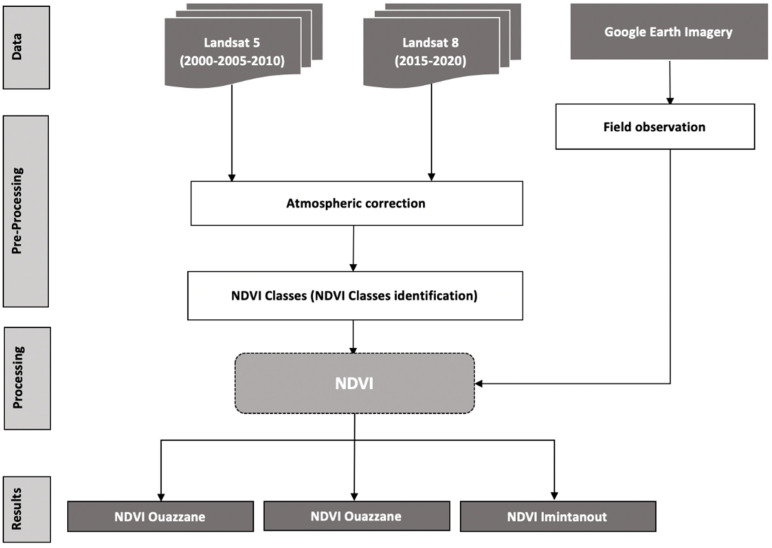
Methodology flowchart.

This work was based on a time series of Landsat 5 and 8 satellite images, which were downloaded from USGS, Earth explorer (NASA), https://earthexplorer.usgs.gov (accessed on 10 May 2021). We used a set of images covering two decades. This allowed us to obtain at least one image every 5 or 6 years from 2000 to 2019, for the whole study area of the three foci; after an atmospheric correction of the images, those from Landsat 5 were used to calculate the NDVI for the years 2000, 2005, and 2010, whereas those from Landsat 8 were used to calculate the NDVI for the years 2015 and 2019 with ArcMap 10.8 software (Casablanca, Morocco). The methodology is summarized in Figure 2.

The NDVI calculation formula is as follows: (NIR − R)/(NIR + R), where NIR is the near-infrared band, and R is the red band.

### 2.7. Statistical Analyses 

The clinical data and the CL data reported between 2000 and 2019 were analyzed using the statistical software R, version 4.1.2 (http://www.R-project.org, accessed on 15 June 2021). The linear regression model was applied to assess the impact of some of the investigated factors. The relationship between the incidence of CL and environmental factors (temperature, rainfall, humidity, wind speed, and vegetation index) was tested using Pearson’s rank correlation, as previously described by [24]. Differences were considered significant when *p* < 0.05. Standardized principal component analysis (PCA) was carried out to generate an integrative description of the data (incidence of the disease and the environmental data) over the years. The incidence of the disease in each year was analyzed using the environmental data of the year before (T − 1), because, according to a normal *Leishmania* cycle of infection, sandflies only start their activities in the summer; thus, counting the incubation time in human hosts, cases tend to appear at the end of the year and are, therefore, counted the year after. Additionally, the clinical data were analyzed using Fisher’s exact test; a comparison between the number of males and females carrying CL lesions, as well as the distribution of lesions (face and upper limbs), was performed. One sample *t*-test was used to calculate the statistically significant differences between the distribution of CL, age groups, and type of lesions. One sample *t*-test was also used to calculate the statistically significant differences between the three diagnostic methods performed in this study. Differences between groups were considered to be statistically significant at *p* < 0.05. 

### 2.8. Parasitological and Molecular Study

The smear slides were analyzed under a microscope with a 100× immersion objective to determine the parasite load. All slides were screened more than once before giving a final result. DNA extraction was performed using swabs after phenol–chloroform extraction followed by ethanol precipitation, as described by [25]. The DNA sample quantification was determined using NanoDrop (Thermo Fisher Scientific, Waltham, MA, USA), adjusting the final concentration of each sample to 50 ng/μL.

For *Leishmania* detection and identification, we used the Nested KDNA-PCR, with two pairs of primers: the forward primer CSB2XF with the reverse primer CSB1XR for the first stage of the reaction, and the forward primer 13Z with the reverse primer LiR for the second stage of the reaction, as described by Noyes et al. [26]. ITS1-PCR was used to study the population structure of *L. tropica* collected in the three areas. The two protocols of PCR were detailed by [11].

Electrophoresis of PCR products was performed on 1.5% and 1% agarose gels for ITS1-PCR and Nested KDNA-PCR, respectively, to which 2 μL of ethidium bromide solution (10 mg/mL) was added (Promega, Madison, WI, USA). The electrophoretic migration was carried out in 0.5× TBE buffer, and the gel was visualized under UV light.

The amplified KDNA fragments on the agarose gel were compared with standard and marker bands (100 bp DNA ladder marker, Bioline) to identify the *Leishmania* species.

Sequencing: the final ITS1-PCR products of about 350 bp were purified using the Exs Pure Enzymatic PCR cleanup kit (NIMAGEN, Nijmegen, The Netherlands), and then sequenced using the BrillantDye Terminator Cycle Sequencing Kit v1.1 (Nimagen, The Netherlands) and ABI PRISM 3130xL DNA automated sequencer (Applied Biosystems, Waltham, MA, USA).

Sequencing data were analyzed using Chromas v.2.6.2 software (Technelysium), aligned, and compared to entries retrieved from Genbank, using the multiple alignment program MEGA7.

### 2.9. Basic Statistics, Tests for Selection, and Phylogeny

Estimates of genetic diversity were assessed using DnaSP v. 4.0 and Arlequin v. 3.5 [27]. The number of segregating sites, the number of haplotypes (H), haplotype diversity, the average number of nucleotide differences, and nucleotide diversity were calculated. To deduce genetic diversity, we used the nuclear gene ITS1. Pairwise FST values were calculated using DnaSP [28]. Three geographic groups were defined for the comparative study: (i) Foum Jemaa samples, (ii) Imintanout samples, and (iii) Ouazzane samples. To assess whether the examined gene evolved randomly or not, Tajima’s D test [29] and Fu’s F test [30] were performed on DnaSP and Arlequin.

To reconstruct the phylogenetic tree, we used MrBayes v. 3.2.1 [31]. In the first step, the optimal substitution model was estimated using MrModeltest v.2.4 [32]. Runs were computed in MrBayes for 200,000 generations while sampling every 100 generations. Then, the tree was visualized and edited using FigTree v.1.4.2 [33]. A haplotype network was constructed and visualized using PopArt [34].

## 3. Results

### 3.1. Impact of Environmental Factors Associated with the Incidence of CL

Environmental factors (represented by temperature, rainfall, humidity, wind speed, and the vegetation index) that are associated with the incidence of CL in the studied CL foci are shown in Table 1.

**Figure 3:**
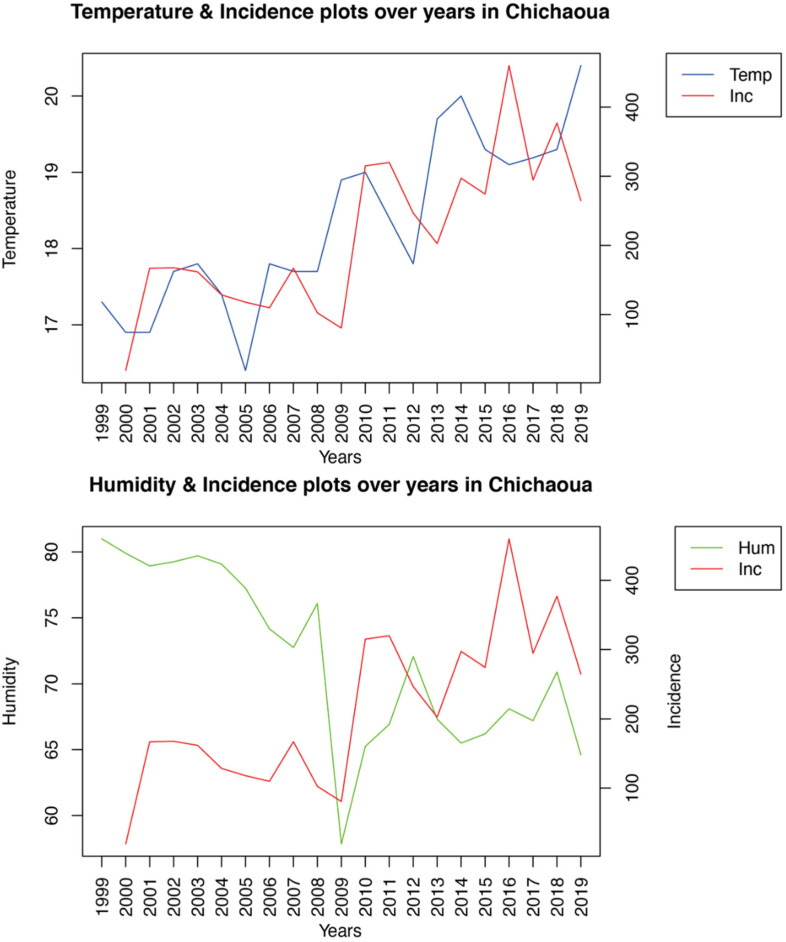
Temperature, humidity, and incidence evolution over the years in Imintanout.

According to statistical analysis, significant Pearson correlations were observed between the incidence of CL in Imintanout and temperature and humidity, while no significant correlations were found between CL incidence and the environmental risk factors in Foum Jemaa and Ouazzane. Moreover, the linear regression correlation confirmed the association between the incidence of CL in Imintanout and temperature (*r*^2^ = 0.6076, df = (1.18), *p*-value = 3.09 × 10^−5^), as well as humidity (*r*^2^ = 0.6306, df = (1.18), *p*-value = 1.77 × 10^−5^), as shown in Table 2 and Figure 3.

**Figure 4:**
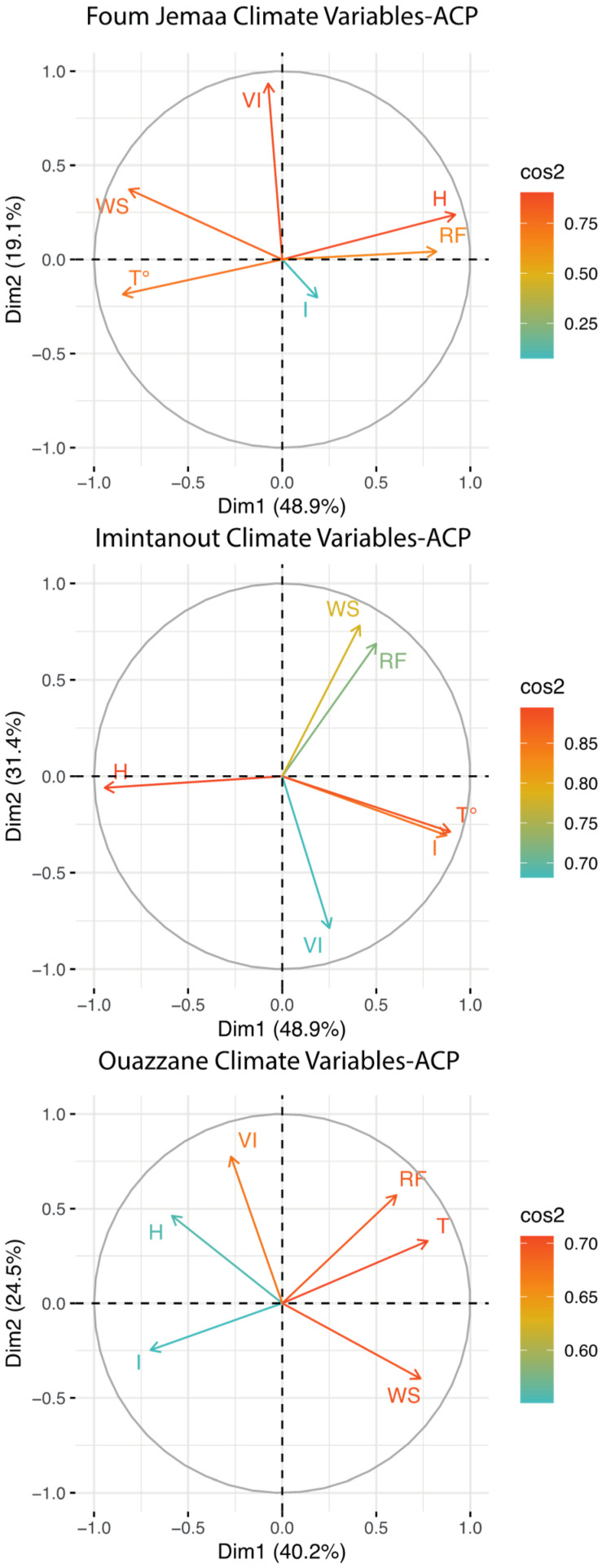
Results of the principal component analysis. I = Incidence of CL; T = temperature; RF = rainfall; H = Humidity; WS = windspeed; VI = vegetation Index.

To generate an integrative description of the data, a PCA was carried out. It resulted in six synthetic variables (PCs), with the first three factors summarizing approximately 86.16%, 89.21%, and 81.02% of the observed variance for Foum Jemaa, Imintanout, and Ouazzane, respectively. Indeed, we can observe in Figure 4 that, in the case of Imintanout, incidence was positively correlated with temperature, while it was negatively correlated with humidity.

### 3.2. Patient Data Analysis 

Among the 80 patients with CL enrolled in this study, 57.5% were females and 42.5% were males (Table 3). We noticed a statistically significant gender difference (*p* = 0.04949229). 

Patients with CL were aged between six months and 70 years, with a median age of 8.7 years (interquartile range: 4.34–13.04 years), and more than half of the patients (57.5%) were no more than 10 years old (Table 3). The difference between the age groups was not statistically significant (t = 2.0239, *p*-value = 0.113).

### 3.3. Clinical Analysis 

The clinical characteristics of the lesions according to type, number, and location in the CL patients are summarized in Table 4.

Overall, 67.5% of CL patients had a single lesion, while 32.5% had multiple lesions. The face was the most affected area, followed by the upper limbs, with an incidence of 81.25% and 13.75%, respectively (Table 4). The difference between these two areas of infection was statistically significant (*p* = 0.0376422).

CL patients presented different clinical forms of lesions. However, the most common form was the papulonodular form, present in 61.25% of CL patients. Furthermore, 37.5% of the patients had ulcero-crusted lesions, while only one patient had an ulcerative lesion (Table 4). The difference between the type of lesions was not statistically significant (t = 1.9107, *p*-value = 0.1962).

### 3.4. Parasitological and Molecular Analysis

Three diagnostic methods were used to confirm the clinical diagnosis of the 80 sampled patients with suspicious CL: direct examination, culture, and PCR amplification of KDNA. The slide smears and *Leishmania* isolation cultures were performed using dermal scraping products; meanwhile, for molecular analysis, cotton swabs were used. We confirmed CL in 67 out of the 80 patients (overall positivity rate: 83.75%) by reference gold-standard diagnosis (detection of amastigotes in Giemsa-stained smears using microscopy and/or culture isolation of *Leishmania*) and/or PCR. The positivity rate of each method of diagnosis used is presented in Table 5. The difference between the diagnostic methods was not statistically significant (t = 3.163, *p*-value = 0.08709).

KDNA amplification also allowed us to identify the *Leishmania* species circulating in each focus. *L. tropica* was found in Foum Jemaa and Imintanout, while both *L. tropica* and *L. infantum* were identified in Ouazzane (Table 6).

### 3.5. Population Structure and Genetic and Haplotype Diversities 

Among the 63 KDNA-positive samples, we selected 35 samples in which we amplified the ITS1 fragments sized 318–325 bp; 25 of them were positive and previously identified as *L. tropica*. In addition to these twenty-five strains, four other strains isolated in Morocco (more precisely, Azilal province) were included in the study; two were isolated from *Phlebotomus sergenti*, one was isolated from a human, and one was isolated from a dog. These sequences were submitted to GenBank under accession numbers OK599037 to OK599065 (for ITS1).

We identified 29 polymorphic sites that led to the definition of 13 haplotypes. One haplotype was found in both the Foum Jemaa (FJ) and Imintanout (IT) populations and had the highest frequency in the total dataset (34.48%). In addition to this haplotype, another haplotype was shared by the three populations. The shared haplotypes represented 67.23% of the total number of individuals. The remaining 11 haplotypes were unique to a single population. Haplotype diversity was large in FJ and IT, ranging from Hd = 0.784 to H = 0.789; however, for Ouazzane, no difference was found, which can be explained by the small sample size. In contrast, nucleotide diversity was relatively low for each population, ranging between 0 and 0.00442 (Table 7).

The FST values were relatively high and showed a high differentiation among the populations, except between Foum Jemaa and Ouazzane (FST = 0.09474) (Table 8).

**Figure 5:**
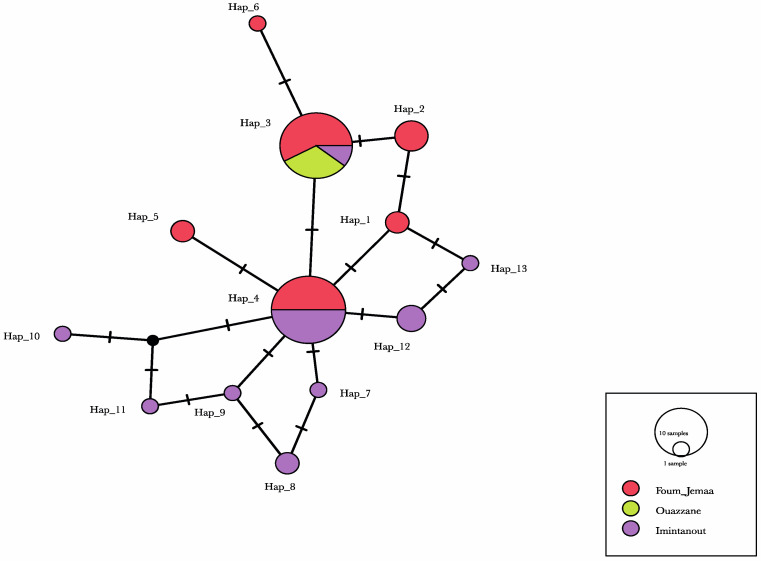
Haplotype network. The circle size is relative to the number of haplotype copies present in each dataset. A black bar represents a single nucleotide change; black dots on branches represent inferred missing haplotypes (single nucleotide changes).

The haplotype network in Figure 5 shows the common haplotypes present in the three provinces, whereas another haplotype was present only in Foum Jemaa and Imintanout, and 11 single haplotypes were shared between FJ and IT. Regarding these populations, the network showed a very high frequency of unique mutations and a low level of sequence divergence, which can be a sign of rapid population expansion.

Tajima’s D test results (Table 8) rejected neutrality for the FJ and IT populations, suggesting a recent population expansion; this was also reflected by the star-like shape of the haplotype network, but the *p*-value for the test was not significant. Only the results of samples from Ouazzane evolved according to a mutation–drift equilibrium with a Tajima’s D of 0; Fu’s F test could not be computed as there was only one allele in the sample (Table 9).

### 3.6. Phylogenetic Analysis

**Figure 6:**
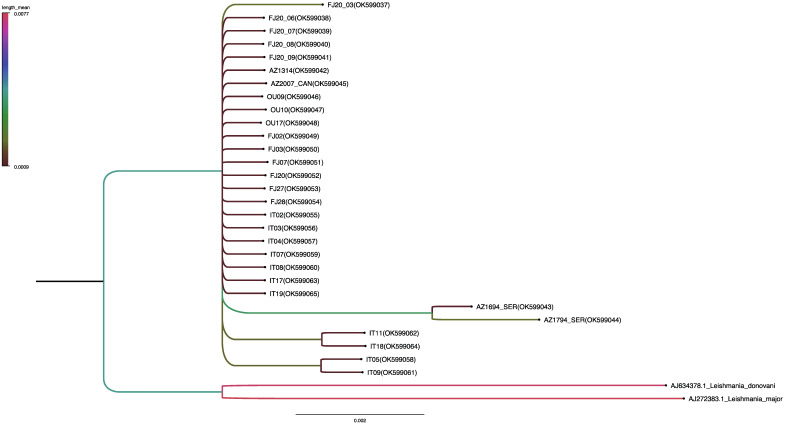
Bayesian tree based on the ITS1 sequence.

A phylogenetic tree was used to lay out the evolutionary biology of *L. tropica* using the nuclear marker ITS1; the likelihood setting from the best-fit model (HKY) selected by AIC in MrModeltest 2.4 was applied. Samples from this study mainly involved patients. All *L. tropica* sequences were grouped into one big clade comprising the strains from the different areas studied. Additional *Leishmania* spp. retrieved from the GenBank database were used as an outgroup. The tree topology showed high similarity among most *L. tropica* isolates. (Figure 6).

## 4. Discussion

Since the first case was reported in 1987 in Azilal Province (Tanant) [5], CL caused by *L. tropica* has spread all over the country, becoming a serious public health problem; it is also the most difficult *Leishmaniasis* form to control due to its wide geographical distribution [13]. The spread of *L. tropica* is probably related to the high ecological plasticity of its vector, *Phlebotomus sergenti* [35].

Our data revealed that children under 10 years old displayed the highest rate of infection (57.5%), in concordance with previously published data on the incidence of CL in other foci throughout Morocco [36] and in other endemic countries, where the majority of infected people were under 16 years old [37,38]. The prevalence of CL is reported to increase generally with age up to 15 years, after which it stabilizes, probably reflecting the progressive buildup of immune protective status [39]. On the other hand, in line with widely known evidence reported for *L. tropica*-induced CL, our results showed that the face was the most affected site, generally with single lesions [40,41], in contrast to *L. major*, which causes multiple lesions generally located on the limbs [42]. Analysis also showed that women were more affected than men, which can be explained by the fact that, during the hot summer nights characterizing these regions, men are known to stay and sleep outdoors (i.e., on balconies or terraces), unlike women, who are often indoors; *P. sergenti* is endophilic and anthropophagic [43]. In addition, the most common form of lesion in our study was the papulonodular form. El Hamouchi, A et al., highlighted that, in the case of CL caused by *L*. *major*, the most frequent clinical lesion form was the ulcero-crusted form [36]. Clinical manifestations of *Leishmania* infections depend on multifactorial parameters, such as human genetic susceptibility and the genetic background of the parasite. Factors related to the vector may also affect CL manifestations [44].

The World Health Organization considers leishmaniasis to be a climate-sensitive disease, occupying a characteristic ‘climate space’ that is strongly affected by changes in rainfall, atmospheric temperature, and humidity [45]. According to various studies, the incidence of leishmaniasis is influenced by a variety of environmental, landscape, and socioeconomic factors [15,46]. Due to the dynamic nature of leishmaniasis as a vector-borne disease, demographic factors and human activity and behaviors are factors that need to be closely monitored [21,47]. CL transmission is related to the various *Leishmania* spp., particularly vector dynamics, which determines the presence of leishmaniasis as a function of climatic and environmental changes [21].

Using a linear regression model, we analyzed the impact of diverse bioclimatic and environmental variables, including mean temperature, annual rainfall, relative humidity, average wind speed, and vegetation index, on the incidence of CL over 20 years. Our results differed according to the foci. Indeed, while temperature and humidity were strongly intercorrelated and significantly associated with the incidence of CL in Imintanout, southwestern Morocco, none of the risk factors studied were significantly correlated with the incidence of the disease in the other two foci: Foum Jemaa and Ouazzane in central and northern Morocco, respectively. Focusing on another aspect of *L. tropica*-induced CL in the southwest of the country, no significant correlation was established between some environmental predictors such as temperature, rainfall, and altitude and the incidence of leishmaniasis [22].

Temperature has been identified as an important risk factor associated with leishmaniasis in Mediterranean, tropical, and arid regions. Indeed, small changes in temperature have a profound impact on the developmental time and metabolism of sandflies, as well as on the *Leishmania* development cycle within its vector [48]. This could potentially affect the distribution of leishmaniasis and allow transmission of the *Leishmania* parasite in areas that were previously free of leishmaniasis [45].

In Ethiopia, a temperature range between 17.2 and 23.8 °C was associated with CL occurrence [49]. In South America, a peak of incidence of CL was found in Chaparral, Colombia, with a mean temperature of 20.6 ± 1.4 °C [50]; in Tunisia, a temperature of 9.4–22.1 °C contributed to 20.7% of the variation in an ecological niche model of the vector [51].

Humidity plays an important role in the survival, development, and activity of sandflies. Indeed, the humidity level during the night influences the growth of flies and, consequently, the occurrence and distribution of leishmaniasis [52]. In Tunisia, it was found that a relative humidity between 30% and 45% increased the ZCL incidence in an *L. major* focus [53]. In Iran, a humidity range between 27% and 30% was registered in the CL hotspot areas in Isfahan [54].

Other environmental and climatic factors have been reported to impact the incidence and distribution of leishmaniasis in different regions of the world. Indeed, in the Andean region of Colombia and in Brazil, annual rainfall is an important risk factor for CL [55,56]. Furthermore, in Iran and Turkey, high NDVI values have been associated with the occurrence of CL [57,58]. 

In Sri Lanka, as in Iran, the wind speed was identified as a predictor, revealing a positive correlation with the incidence of leishmaniasis [59,60]. However, a negative association was demonstrated between maximum wind speed, rainfall, altitude, and vegetation cover and CL incidence in Iran [61]. In contrast to these studies, the analysis of our data revealed that vegetation index, precipitation, and wind speed had no impact on the incidence of CL in the three study sites.

Around the world, many studies have tried to find an association between climatic and environmental factors and the occurrence of leishmaniasis; some have been able to find associations, while others have not been able to draw any significant conclusions. This discord can be explained by the fact that leishmaniasis is a multifactorial disease [15], and climatic factors are not the only ones influencing the occurrence of the disease; other factors need to be monitored, such as demographic and social factors, as well as the density of the vector and the matter of hygiene, which is generally neglected in rural areas. The presence of waste or stables in the vicinity of houses, as well as the use of traditional building materials for house construction, are all factors that favor the development of the vector and reservoirs, resulting in an increase in leishmaniasis cases.

In addition to elucidating the climatic and environmental factors that may influence the distribution of CL, we tried to shed light on the population structure of the *L. tropica* parasite isolated in the three geographically distant CL foci. 

Our results showed low to medium geographic differentiation among the *L. tropica* populations, using pairwise differentiation. The molecular diversity index values were roughly similar among the Foum Jemaa and Imintanout populations, in contrast to the Ouazzane strains. Indeed, in the latter foci, only one single haplotype was identified among the three strains collected in this region. This should be explored with a larger sample.

Haplotype diversity ranged between 0.784 and 0.789, with a total of 13 haplotypes. The analysis of ITS1-5.8S rRNA gene and ITS2 sequences from 31 *P. sergenti* revealed a great heterogeneity of *L. tropica* isolated from *P. sergenti*, segregated into 16 haplotypes with phylogenetic relatedness to Indian strains and one Moroccan strain isolated from a CL patient [62].

By analyzing a combination of mitochondrial and nucleic genes, Fotouhi-Ardakani, et al. [63], demonstrated that *L. tropica* has high haplotype diversity. An important haplotype diversity was also highlighted by Arroub et al. [42], who analyzed the internal transcribed spacer ITS1 and 5.8S rDNA gene sequences of *L. tropica* isolated from Moroccan patients. Recently, El Kacem et al. [64], confirmed the high intraspecific variability of *L. tropica* in Morocco using the MLST approach; moreover, the MLST analysis allowed for a distinct separation of *L. tropica* strains according to their geographical origin.

While haplotype diversity was high in our study, low nucleotide diversity values indicated only small differences between haplotypes. This was also clear when examining the minimum-spanning haplotype network, which mostly showed single-nucleotide differences between haplotypes.

Regarding the population differentiation, the Foum Jemaa and Imintanout populations and the Ouazzane and Imintanout populations are remotely related to each other, as indicated by the high and significant FST value.

To evaluate the population expansion of *L. tropica*, multiple selective neutrality tests were performed. Statistical tests originally developed to test the selective neutrality of a mutation were implemented [65]. We selected two tests that are frequently used to detect population growth and vary somewhat in their approach. Tajima’s D test [29] is a common way to quantify the demographic signatures of population growth on the basis of the distribution of allele frequency of segregating nucleotide sites. A positive value points to a population with a deficiency of rare haplotypes that has experienced a recent bottleneck, whereas a negative value indicates a bias toward rare alleles, with the latter being a signature of recent population expansion. Fu’s F test is based on the distribution of alleles or haplotypes, with negative values indicating possible recent population growth, while positive values are evidence of allele deficiency, as would be expected from a recent population bottleneck [30]. In our study, Tajima’s D test showed nonsignificant negative values for the *L. tropica* population, except for the Ouazzane population, which showed a null test value. Furthermore, Fu’s F test gave nonsignificant positive values for all populations except for Ouazzane’s *L. tropica*, represented by a single allele, which prevented results from being inferred.

The negative values obtained using Tajima’s D test signify an excess of low-frequency polymorphisms relative to expectation; on the other hand, the positive values obtained using Fu’s F test are evidence of allele deficiency, as would be expected from a recent population bottleneck. However, overall, in all populations, Tajima’s D and Fu’s F test statistics were not statistically significant, indicating consistency with a population at a drift–mutation equilibrium. 

Further analysis, including additional DNA markers and a larger sample size, could provide a more complete perspective on *L. tropica’s* population structure in these three regions.

## 5. Conclusions

The impact of climatic and environmental factors on the incidence of CL due to *L. tropica* differs between foci. Indeed, while a relationship between the increased risk of occurrence of this CL form and climatic factors was found in Imintanout in southwestern Morocco, no direct relationship was found in the other studied foci. Further research analyzing the interactions of risks factors and how they vary according to vectors, reservoir breeds, and environmental conditions is needed; furthermore, a better understanding of the likely impact of future climatic conditions on the transmission cycle is also required, thus enabling the development of effective control measures.

Early warning remains a high research priority to improve the response of CL control programs in the absence of a safe and effective vaccine.

The molecular analysis performed contributes generally to the knowledge of the current genetic status of *L. tropica* in the three foci studied, pending the expansion of sample sizes to provide a more complete perspective of how this *Leishmania* species is distributed and how it is expanding.

### Study Limitations

The findings of this study must be seen in the light of some limitations. The sample size for the study of the *L*. *tropica* population structure was not sufficient for solid conclusions, especially for the Ouazzane focus. However, we were able to gain insight into the population structures in the three foci and present a hypothesis about population evolution based on the identified haplotypes. 

On the other hand, and for an accurate assessment of the impact of climatic and environmental factors on the incidence of cutaneous leishmaniasis, we encountered certain limitations due to the lack of data on soil types, slopes, the presence of planes of water, housing types, or the socio-economic status in each locality. These limitations deserve to be investigated in future research, in addition to those addressed in this work.

## Figures and Tables

**Table 1:** Correlation matrix between incidence of CL and environmental risk factors in the three CL endemic areas.

**Foum Jemaa**		**Incidence**	**Humidity**	**Rainfall**	**Temperature**	**Wind Speed**	**Vegetation Index**
Incidence	Pearson Correlation	1					
	*p*-Value						
Humidity	Pearson Correlation	0.180	1				
	*p*-Value	0.4475					
Rainfall	Pearson Correlation	0.167	0.662 *	1			
	*p*-Value	0.481	0.001474				
Temperature	Pearson Correlation	0.133	−0.829 *	−0.559 *	1		
	*p*-Value	0.5773	6.026 × 10^−6^	0.01032			
Wind speed	Pearson Correlation	−0.212	−0.629 *	−0.580 *	0.543 *	1	
	*p*-Value	0.3695	0.002968	0.007314	0.01324		
Vegetation index	Pearson Correlation	0.025	0.132	−0.005	0.019	0.306	1
	*p*-Value	0.9136	0.578	0.9804	0.935	0.1892	
**Imintanout**		**Incidence**	**Humidity**	**Rainfall**	**Temperature**	**Wind Speed**	**Vegetation Index**
Incidence	Pearson Correlation	1					
	*p*-Value						
Humidity	Pearson Correlation	−0.806 *	1				
	*p*-Value	1.765 × 10^−5^					
Rainfall	Pearson Correlation	0.167	−0.415	1			
	*p*-Value	0.4819	0.06855				
Temperature	Pearson Correlation	0.793 *	−0.809 *	0.260	1		
	*p*-Value	3.086 × 10^−5^	1.547 × 10^−5^	0.2673			
Wind speed	Pearson Correlation	0.109	−0.407	0.638 *	0.101	1	
	*p*-Value	0.6461	0.07451	0.002461	0.6714		
Vegetation index	Pearson Correlation	0.370	−0.090	−0.265	0.403	−0.368	1
	*p*-Value	0.1083	0.7051	0.2579	0.0777	0.1101	
**Ouazzane**		**Incidence**	**Humidity**	**Rainfall**	**Temperature**	**Wind Speed**	**Vegetation Index**
Incidence	Pearson Correlation	1					
	*p*-Value						
Humidity	Pearson Correlation	0.268	1				
	*p*-Value	0.4252					
Rainfall	Pearson Correlation	−0.375	0.146	1			
	*p*-Value	0.2552	0.6677				
Temperature	Pearson Correlation	−0.469	−0.449	0.549	1		
	*p*-Value	0.1455	0.1656	0.07978			
Wind speed	Pearson Correlation	−0.330	−0.438	0.387	0.243	1	
	*p*-Value	0.321	0.1772	0.2395	0.4712		
Vegetation index	Pearson Correlation	−0.003	0.222	0.102	0.036	−0.472	1
	*p*-Value	0.9925	0.5114	0.7648	0.9152	0.1421	

* *p* < 0.05.

**Table 2:** Results of linear regression between incidence of CL and environmental risk factors.

Regions	Variable	Coefficient	T-Statistic	*p*-Value
Foum Jemaa	Intercept	−52.645	−0.229	0.821
	Humidity	3.224	0.777	0.448
		Adjusted *R*^2^ = −0.02134	F_(1, 18)_ = 0.603	F_(1, 18)_ = 0.4475
	Intercept	65.3075	0.759	0.458
	Rainfall	0.1766	0.720	0.481
		Adjusted *R*^2^ = −0.02604	F_(1, 18)_ = 0.5179	F_(1, 18)_ = 0.481
	Intercept	−134.28	−0.294	0.772
	Temperature	12.89	0.568	0.577
		Adjusted *R*^2^ = −0.03699	F_(1, 18)_ = 0.3222	F_(1, 18)_ = 0.5773
	Intercept	271.42	1.677	0.111
	Wind speed	−21.53	−0.920	0.370
		Adjusted *R*^2^ = −0.008106	F_(1, 18)_ = 0.8472	F_(1, 18)_ = 0.3695
	Intercept	−11.293	−0.009	0.993
	Vegetation index	1.507	0.110	0.914
		Adjusted *R*^2^ = −0.05485	F_(1, 18)_ = 0.01211	F_(1, 18)_ = 0.9136
Imintanout	Intercept	1204.23	7.002	1.55 × 10^−6^ ***
	Humidity	−13.71	−5.782	1.77 × 10^−5^ ***
		Adjusted *R*^2^ = 0.6306	F_(1, 18)_ = 33.44	F_(1, 18)_ = 1.765 × 10^−5^
	Intercept	163.9054	2.225	0.0391 *
	Rainfall	0.1643	0.718	0.4819
		Adjusted *R*^2^ = −0.02616	F_(1, 18)_ = 0.5156	F_(1, 18)_ = 0.4819
	Intercept	−1343.14	−4.751	0.00016 ***
	Temperature	85.47	5.515	3.09 × 10^−5^ ***
		Adjusted *R*^2^ = 0.6076	F_(1, 18)_ = 30.42	F_(1, 18)_ = 3.086 × 10^−5^
	Intercept	160.233	1.368	0.188
	Wind speed	3.005	0.467	0.646
		Adjusted *R*^2^ = −0.04292	F_(1, 18)_ = 0.2181	F_(1, 18)_ = 0.6461
	Intercept	−420.896	−1.119	0.278
	Vegetation index	6.621	1.690	0.108
		Adjusted *R*^2^ = 0.08895	F_(1, 18)_ = 2.855	F_(1, 18)_ = 0.1083
Ouazzane	Intercept	−225.889	−0.679	0.514
	Humidity	4.116	0.835	0.425
		Adjusted *R*^2^ = −0.03118	F_(1, 9)_ = 0.6976	F_(1, 9)_ = 0.4252
	Intercept	82.933315	3.062	0.0135
	Rainfall	−0.05090	−1.215	0.2552
		Adjusted *R*^2^ = 0.04548	F_(1, 9)_ = 1.476	F_(1, 9)_ = 0.2552
	Intercept	593.00	1.746	0.115
	Temperature	−28.26	−1.594	0.145
		Adjusted *R*^2^ = 0.1335	F_(1, 9)_ = 2.54	F_(1, 9)_ = 0.1455
	Intercept	245.04	1.331	0.216
	Wind speed	−11.78	−1.050	0.321
		Adjusted *R*^2^ = 0.0102	F_(1, 9)_ = 1.103	F_(1, 9)_ = 0.321
	Intercept	52.999215	0.479	0.643
	Vegetation index	−0.00883	−0.010	0.992
		Adjusted *R*^2^ = −0.1111	F_(1, 9)_ = 9.386 × 10^−5^	F_(1, 9)_ = 0.9925

* *p* < 0.05; *** *p* < 0.01.

**Table 3:** Distribution of CL patients by age group and sex.

Age Group (Years)	Sex		Total
	Male	Female	
0–10	21 (26.25%)	25 (31.25%)	46 (57.5%)
11–20	8 (10.0%)	9 (11.25%)	17 (21.25%)
21–30	1 (1.25%)	3 (3.75%)	4 (5.0%)
31–40	2 (2.5%)	1 (1.25%)	3 (3.75%)
41–70	2 (2.5%)	8 (10.0%)	10 (12.5%)
Total	34 (42.5%)	46 (57.5%)	80 (100.0%)

**Table 4:** Clinical characteristics of CL patients (*n* = 80). The sum of the distribution of the lesions is not equal to the total number, owing to the presence of more than lesion in some patients.

Type of Lesion	Number of Patients	(%)
Ulcero-crusted	30	37.5
Papulonodular	49	61.25
Ulcers	1	1.25
Number of lesions		
1	54	67.5
2–5	25	31.25
≥5	1	1.25
Distribution of lesions		
Face	65	81.25
Upper limbs	11	13.75
Lower limbs	7	8.75

**Table 5:** Positivity rates of the three diagnostic methods used for the 80 sampled patients.

	Positive	Negative	Positivity Rate (%)
Microscopy	57	23	71.25%
KDNA-PCR	63	17	78.75%
In vitro culture	17	63	21.25%
Parasitological techniques and/or PCR *	67	13	83.75%

* Parasitological techniques = microscopy and/or in vitro culture; PCR = KDNA-PCR.

**Table 6:** Results of Nested KDNA-PCR for CL patients.

Number of CL Patients per Region		Nested KDNA-PCR	
		Positive	Negative
Foum Jemaa	40	33 *L. tropica*	7
Imintanout	20	16 *L. tropica*	4
Ouazzane	20	14 (9 *L. tropica*, 5 *L. infantum*)	6
Total	80	63	17

**Table 7:** Sample size (*N*), number of polymorphic sites (PS), number of haplotypes (H), haplotype diversity (Hd), nucleotide differences (K), and nucleotide diversity (Pi) per population.

	*N*	PS	H	Hd	K	PI
Foum Jemaa	30	5	7	0.789	1.280	0.00404
Ouazzane	6	0	1	0	0	0
Imintanout	22	7	9	0.784	1.398	0.00442

**Table 8:** Comparative FST between *L. tropica* populations.

	Foum Jemaa	Ouazzane	Imintanout
Foum Jemaa			*
Ouazzane	0.09474		*
Imintanout	0.21116	0.25357	

* Significant *p*-value.

**Table 9:** Results of Tajima’s D and Fu’s F neutrality tests.

	Foum Jemaa	Ouazzane	Imintanout
Tajima’s D test	−0.19883	0.00000	−0.86988
*p*-Value	0.45100	1.00000	0.20600
Fu’s F test	1.15300	N/A	0.52114
*p*-Value	0.76700	N/A	0.62600

## Data Availability

Some of the meteorological data were obtained from https://TuTiempo.net (accessed on 5 April 2021) (powered by Tutiempo Network, S.L., Madrid, Spain). The NDVI calculations were based on a time series of Landsat 5 and 8 satellite images, which were downloaded from USGS, Earth explorer (NASA), https://earthexplorer.usgs.gov/ (accessed on 10 May 2021).

## Supplementary Materials

The following supporting information can be downloaded at: https://www.mdpi.com/article/10.3390/microorganisms10091712/s1; Table S1: The annual incidence rate of CL in each province: incidence rate = (total number of CL cases per year/total population at risk) × 100,000.

## References

[1] Laboudi M., Sahibi H., Elabandouni M., Nhammi H., Ait Hamou S., Sadak A. (2018). A review of cutaneous leishmaniasis in Morocco: A vertical analysisto determine appropriate interventions for control and prevention. Acta Trop..

[2] WHO Leishmaniasis Fact-Sheet. https://www.who.int/news-room/fact-sheets/detail/leishmaniasis.

[3] El Mazini S., Ejghal R., Bekhti K., Lemrani M. (2021). The Sporadic cutaneous leishmaniasis due to Leishmania infantum in Morocco: A presumably trend towards endemicity. Acta Trop..

[4] Ajaoud M., Es-sette N., Hamdi S., El-Idrissi A.L., Riyad M., Lemrani M. (2013). Detection and molecular typing of Leishmania tropica from Phlebotomus sergenti and lesions of cutaneous leishmaniasis in an emerging focus of Morocco. Parasit. Vectors.

[5] Marty P., Le Fichoux Y., Pratlong F., Rioux J.A., Rostain G., Lacour J.P. (1989). Cutaneous leishmaniasis due to Leishmania tropica in a young Moroccan child observed in Nice, France. Trans. R. Soc. Trop. Med. Hyg..

[6] Pratlong F., Rioux J.A., Dereure J., Mahjour J., Gallego M., Guilvard E., Lanotte G., Perieres J., Martini A., Saddiki A. (1991). Leishmania tropica in Morocco. IV--Intrafocal enzyme diversity. Ann. Parasitol. Hum. Comp..

[7] Guessous-Idrissi N., Chiheb S., Hamdani A., Riyad M., Bichichi M., Hamdani S., Krimech A. (1997). Cutaneous leishmaniasis: An emerging epidemic focus of Leishmania tropica in north Morocco. Trans. R. Soc. Trop. Med. Hyg..

[8] Rhajaoui M., Fellah H., Pratlong F., Dedet J.P., Lyagoubi M. (2004). Leishmaniasis due to Leishmania tropica MON-102 in a new Moroccan focus. Trans. R. Soc. Trop. Med. Hyg..

[9] Guernaoui S., Boumezzough A., Pesson B., Pichon G. (2005). Entomological investigations in Chichaoua: An emerging epidemic focus of cutaneous leishmaniasis in Morocco. J. Med. Entomol..

[10] Fatima A., Faiza S., Hajiba F., Francine P., Dedet J.P., Bouchra E.M., Asmae H., Bouchra D., Khalid H., Abderrahim S. (2015). Epidemiological characteristics of a new focus of cutaneous leishmaniasis caused by Leishmania tropica in Settat, Morocco. Acta Trop..

[11] Daoui O., Ait Kbaich M., Mhaidi I., El Kacem S., Hjiyej Andaloussi L., Akarid K., Lemrani M. (2020). The role of sampling by cotton swab in the molecular diagnosis of cutaneous leishmaniasis. Transbound. Emerg. Dis..

[12] Ait Kbaich M., Mhaidi I., Ezzahidi A., Dersi N., El Hamouchi A., Riyad M., Akarid K., Lemrani M. (2017). New epidemiological pattern of cutaneous leishmaniasis in two pre-Saharan arid provinces, southern Morocco. Acta Trop..

[13] El Idrissi Saik I., Benlabsir C., Fellah H., Lemrani M., Riyad M. (2022). Transmission patterns of Leishmania tropica around the Mediterranean basin: Could Morocco be impacted by a zoonotic spillover?. PLoS Negl. Trop. Dis..

[14] Purse B.V., Masante D., Golding N., Pigott D., Day J.C., Ibañez-Bernal S., Kolb M., Jones L. (2017). How will climate change pathways and mitigation options alter incidence of vector-borne diseases? A framework for leishmaniasis in South and Meso-America. PLoS ONE.

[15] Valero N.N.H., Uriarte M. (2020). Environmental and socioeconomic risk factors associated with visceral and cutaneous leishmaniasis: A systematic review. Parasitol. Res..

[16] Wade T.G., Riitters K.H., Wickham J.D., Jones K.B. (2003). Distribution and causes of global forest fragmentation. Conserv. Ecol..

[17] Cardim M.F.M., Rodas L.A.C., Dibo M.R., Guirado M.M., Oliveira A.M., Chiaravalloti Neto F. (2013). Introduction and expansion of human American visceral leishmaniasis in the state of Sao Paulo, Brazil, 1999–2011. Rev. Saude Publica.

[18] dos Santos Afonso M.M., de Miranda Chaves S.A., Magalhães M.d.A.F.M., Gracie R., Azevedo C., de Carvalho B.M., Rangel E.F. (2017). Ecoepidemiology of American visceral leishmaniasis in Tocantins State, Brazil: Factors associated with the occurrence and spreading of the vector Lutzomyia (Lutzomyia) longipalpis (Lutz & Neiva, 1912) (Diptera: Psychodidae: Phlebotominae). The Epidemiology and Ecology of Leishmaniasis.

[19] Gutierrez J.D., Martínez-Vega R., Ramoni-Perazzi J., Diaz-Quijano F.A., Gutiérrez R., Ruiz F.J., Botello H.A., Gil M., González J., Palencia M. (2017). Environmental and socio-economic determinants associated with the occurrence of cutaneous leishmaniasis in the northeast of Colombia. Trans. R. Soc. Trop. Med. Hyg..

[20] Oliveira A.M., López R.V.M., Dibo M.R., Rodas L.A.C., Guirado M.M., Chiaravalloti-Neto F. (2018). Dispersion of Lutzomyia longipalpis and expansion of visceral leishmaniasis in São Paulo State, Brazil: Identification of associated factors through survival analysis. Parasites Vectors.

[21] Cardenas R., Sandoval C.M., Rodríguez-Morales A.J., Franco-Paredes C. (2006). Impact of climate variability in the occurrence of leishmaniasis in northeastern Colombia. Am. J. Trop. Med. Hyg..

[22] El Alem M.M.M., Hakkour M., Hmamouch A., Halhali M., Delouane B., Habbari K., Fellah H., Sadak A., Sebti F. (2018). Risk factors and prediction analysis of cutaneous leishmaniasis due to Leishmania tropica in Southwestern Morocco. Infect. Genet. Evol..

[23] Arroub H., Alaoui A., Lemrani M., Habbari K. (2012). Cutaneous Leishmaniasis in Foum Jamâa (Azilal, Morocco): Micro-Environmental and Socio-Economical Risk Factors. J. Agric. Soc. Sci..

[24] Mukaka M.M. (2012). Statistics corner: A guide to appropriate use of correlation coefficient in medical research. Malawi Med. J..

[25] van Eys G.J., Schoone G.J., Kroon N.C., Ebeling S.B. (1992). Sequence analysis of small subunit ribosomal RNA genes and its use for detection and identification of Leishmania parasites. Mol. Biochem. Parasitol..

[26] Noyes H.A., Reyburn H., Bailey J.W., Smith D. (1998). A nested-PCR-based schizodeme method for identifying Leishmania kinetoplast minicircle classes directly from clinical samples and its application to the study of the epidemiology of Leishmania tropica in Pakistan. J. Clin. Microbiol..

[27] Excoffier L., Lischer H.E. (2010). Arlequin suite ver 3.5: A new series of programs to perform population genetics analyses under Linux and Windows. Mol. Ecol. Resour..

[28] Nosil P., Funk D.J., Ortiz-Barrientos D. (2009). Divergent selection and heterogeneous genomic divergence. Mol. Ecol..

[29] Tajima F. (1989). Statistical method for testing the neutral mutation hypothesis by DNA polymorphism. Genetics.

[30] Fu Y.X. (1997). Statistical tests of neutrality of mutations against population growth, hitchhiking and background selection. Genetics.

[31] Ronquist F., Huelsenbeck J.P. (2003). MrBayes 3: Bayesian phylogenetic inference under mixed models. Bioinformatics.

[32] Darriba D., Taboada G.L., Doallo R., Posada D. (2012). jModelTest 2: More models, new heuristics and parallel computing. Nat. Methods.

[33] Rambaut A. (2012). FigTree v1. 4. http://tree.bio.ed.ac.uk/software/figtree/.

[34] Leigh J.W., Bryant D. (2015). POPART: Full-feature software for haplotype network construction. Methods Ecol. Evol..

[35] Boussaa S., Pesson B., Boumezzough A. (2009). Faunistic study of the sandflies (Diptera: Psychodidae) in an emerging focus of cutaneous leishmaniasis in Al Haouz province, Morocco. Ann. Trop. Med. Parasitol..

[36] El Hamouchi A., Daoui O., Ait Kbaich M., Mhaidi I., El Kacem S., Guizani I., Sarih M., Lemrani M. (2019). Epidemiological features of a recent zoonotic cutaneous leishmaniasis outbreak in Zagora province, southern Morocco. PLoS Negl. Trop. Dis..

[37] Fakhar M., Pazoki Ghohe H., Rasooli S.A., Karamian M., Mohib A.S., Ziaei Hezarjaribi H., Pagheh A.S., Ghatee M.A. (2016). Genetic diversity of Leishmania tropica strains isolated from clinical forms of cutaneous leishmaniasis in rural districts of Herat province, Western Afghanistan, based on ITS1-rDNA. Infect. Genet. Evol..

[38] Charyyeva A., Çetinkaya Ü., Özkan B., Şahin S., Yaprak N., Şahin I., Yurchenko V., Kostygov A.Y. (2021). Genetic diversity of Leishmania tropica: Unexpectedly complex distribution pattern. Acta Trop..

[39] Reithinger R., Dujardin J.C., Louzir H., Pirmez C., Alexander B., Brooker S. (2007). Cutaneous leishmaniasis. Lancet Infect Dis..

[40] Aoun K., Bouratbine A. (2014). Cutaneous leishmaniasis in North Africa: A review. Parasite.

[41] Arroub H., Hamdi S., Ajaoud M., Habbari K., Lemrani M. (2013). Epidemiologic study and molecular detection of Leishmania and sand fly species responsible of cutaneous leishmaniasis in Foum Jamâa (Azilal, Atlas of Morocco). Acta Trop..

[42] El Hamouchi A., Ajaoud M., Arroub H., Charrel R., Lemrani M. (2019). Genetic diversity of Leishmania tropica in Morocco: Does the dominance of one haplotype signify its fitness in both predominantly anthropophilic Phlebotomus sergenti and human beings?. Transbound. Emerg. Dis..

[43] Arroub H., Alaoui A., El Miri H., Lemrani M., Habbari K. (2012). Spatiotemporal Distribution of Phlebotomine Sand Flies (Diptera: Psychodidae) in a Focus of Cutaneous Leishmaniasis in Foum Jamâa (Azilal, Atlas of Morocco). J. Life Sci..

[44] Banuls A.L., Bastien P., Pomares C., Arevalo J., Fisa R., Hide M. (2011). Clinical pleiomorphism in human leishmaniases, with special mention of asymptomatic infection. Clin. Microbiol. Infect..

[45] Who E.C. (2010). Control of the leishmaniases. World Health Organ. Tech. Rep. Ser..

[46] Oryan A., Akbari M. (2016). Worldwide risk factors in leishmaniasis. Asian Pac. J. Trop. Med..

[47] Ghatee M.A., Haghdoost A.A., Kooreshnia F., Kanannejad Z., Parisaie Z., Karamian M., Moshfe A. (2018). Role of environmental, climatic risk factors and livestock animals on the occurrence of cutaneous leishmaniasis in newly emerging focus in Iran. J. Infect Public Health.

[48] Kholoud K., Denis S., Lahouari B., El Hidan M.A., Souad B. (2018). Management of Leishmaniases in the Era of Climate Change in Morocco. Int. J. Environ. Res. Public Health.

[49] Seid A., Gadisa E., Tsegaw T., Abera A., Teshome A., Mulugeta A., Herrero M., Argaw D., Jorge A., Kebede A. (2014). Risk map for cutaneous leishmaniasis in Ethiopia based on environmental factors as revealed by geographical information systems and statistics. Geospat. Health.

[50] Valderrama-Ardila C., Alexander N., Ferro C., Cadena H., Marín D., Holford T.R., Munstermann L.E., Ocampo C.B. (2010). Environmental risk factors for the incidence of American cutaneous leishmaniasis in a sub-Andean zone of Colombia (Chaparral, Tolima). Am. J. Trop. Med. Hyg..

[51] Chalghaf B., Chlif S., Mayala B., Ghawar W., Bettaieb J., Harrabi M., Benie G.B., Michael E., Salah A.B. (2016). Ecological niche modeling for the prediction of the geographic distribution of cutaneous leishmaniasis in Tunisia. Am. J. Trop. Med. Hyg..

[52] Rajesh K., Sanjay K. (2013). Change in global climate and prevalence of visceral leishmaniasis. Int. J. Sci. Res. Publ..

[53] Talmoudi K., Bellali H., Ben-Alaya N., Saez M., Malouche D., Chahed M.K. (2017). Modeling zoonotic cutaneous leishmaniasis incidence in central Tunisia from 2009-2015: Forecasting models using climate variables as predictors. PLoS Negl. Trop. Dis..

[54] Ramezankhani R., Sajjadi N., Nezakati Esmaeilzadeh R., Jozi S.A., Shirzadi M.R. (2017). Spatial analysis of cutaneous leishmaniasis in an endemic area of Iran based on environmental factors. Geospat. Health.

[55] Karagiannis-Voules D.A., Scholte R.G., Guimarães L.H., Utzinger J., Vounatsou P. (2013). Bayesian geostatistical modeling of leishmaniasis incidence in Brazil. PLoS Negl. Trop. Dis..

[56] Pérez-Flórez M., Ocampo C.B., Valderrama-Ardila C., Alexander N. (2016). Spatial modeling of cutaneous leishmaniasis in the Andean region of Colombia. Memórias Inst. Oswaldo Cruz.

[57] Kavur H., Artun O. (2017). Geographical Information Systems in Determination of Cutaneous Leishmaniasis Spatial Risk Level Based on Distribution of Vector Species in Imamoglu Province, Adana. J. Med. Entomol..

[58] Shiravand B., Tafti A.A.D., Hanafi-Bojd A.A., Almodaresi S.A., Mirzaei M., Abai M.R. (2018). Modeling spatial risk of zoonotic cutaneous leishmaniasis in Central Iran. Acta Trop..

[59] Ramezankhani R., Hosseini A., Sajjadi N., Khoshabi M., Ramezankhani A. (2017). Environmental risk factors for the incidence of cutaneous leishmaniasis in an endemic area of Iran: A GIS-based approach. Spat. Spatiotemporal Epidemiol..

[60] Galgamuwa L.S., Dharmaratne S.D., Iddawela D. (2018). Leishmaniasis in Sri Lanka: Spatial distribution and seasonal variations from 2009 to 2016. Parasit. Vectors.

[61] Ramezankhani R., Sajjadi N., Jozi S.A., Shirzadi M.R. (2018). Climate and environmental factors affecting the incidence of cutaneous leishmaniasis in Isfahan, Iran. Environ. Sci. Pollut. Res..

[62] Ajaoud M., Es-Sette N., Charrel R.N., Laamrani-Idrissi A., Nhammi H., Riyad M., Lemrani M. (2015). Phlebotomus sergenti in a cutaneous leishmaniasis focus in Azilal province (High Atlas, Morocco): Molecular detection and genotyping of Leishmania tropica, and feeding behavior. PLoS Negl. Trop. Dis..

[63] Fotouhi-Ardakani R., Dabiri S., Ajdari S., Alimohammadian M.H., AlaeeNovin E., Taleshi N., Parvizi P. (2016). Assessment of nuclear and mitochondrial genes in precise identification and analysis of genetic polymorphisms for the evaluation of Leishmania parasites. Infect. Genet. Evol..

[64] El Kacem S., Kbaich M.A., Daoui O., Charoute H., Mhaidi I., Ejghal R., Barhoumi M., Guizani I., Bennani H., Lemrani M. (2021). Multilocus sequence analysis provides new insight into population structure and genetic diversity of Leishmania tropica in Morocco. Infect. Genet. Evol..

[65] Ramos-Onsins S.E., Rozas J. (2002). Statistical properties of new neutrality tests against population growth. Mol. Biol. Evol..

