# Supplementary material for: Environmental, Climatic, and Parasite Molecular Factors Impacting the Incidence of Cutaneous Leishmaniasis Due to Leishmania tropica in Three Moroccan Foci
Source: Microorganisms. 2022 Aug 25;10(9):1712. doi: 10.3390/microorganisms10091712 (PMC9506065; doi:10.3390/microorganisms10091712)
Supplement: Supplementary file 1 [file microorganisms-10-01712-s001.zip › microorganisms-1862400-supplementary.pdf]

Table S1 : The annual incidence rate of CL in each province: incidence rate = (total number of CL cases per year/total population at risk) × 100,000.

Localities/Incidences	2000	2001	2002	2003	2004	2005	2006	2007	2008	2009	2010	2011	2012	2013	2014	2015	2016	2017	2018	2019
Foum Jemaa	3,61	17,79	13	18,8	16,8	21,65	45,1	84,7	104	146,1	495	248,5	209,68	186,19	158,7	131,9	175,6	94,7	156,7	158,5
Imintanout	18,96	166,9	167,6	161,7	128,38	117,8	109,7	167	102,4	80,4	315	319,9	246,7	202,59	297,2	274,2	460,1	294,5	377,1	264,5
Ouazzane	N/A	N/A	N/A	N/A	N/A	N/A	N/A	N/A	N/A	9,42	17,36	60,5	53,8	60,76	46,9	36	59,87	23,14	91,3	112,2

for Ouazzane, the period was shorter (2009 to 2019) due to a lack of information, justified by the fact that CL cases in Ouazzane were very low and were, therefore, added to the closest province to Ouazzane.
